# Factors associated with prolonged hospitalization of patients with corona virus disease (COVID-19) in Uganda: a retrospective cohort study

**DOI:** 10.1186/s41182-022-00491-8

**Published:** 2022-12-28

**Authors:** Prossie M. Ingabire, Ritah Nantale, Quraish Sserwanja, Susan Nakireka, Milton W. Musaba, Asad Muyinda, Criscent Tumuhaise, Edith Namulema, Felix Bongomin, Agnes Napyo, Rozen Ainembabazi, Ronald Olum, Ian Munabi, Sarah Kiguli, David Mukunya

**Affiliations:** 1 Department of Medicine, Nsambya Hospital, Kampala, Uganda; 2 Department of Nursing, Busitema University, Tororo, Uganda; 3 Department of Programmes, GOAL, Arkaweet Block 65 House No. 227, Khartoum, Sudan; 4 Department of Medicine, Mengo Hospital, Kampala, Uganda; 5 Department of Medicine and Dentistry, Uganda Christian University, Kampala, Uganda; 6 Department of Obstetrics and Gynaecology, Mbale Regional Referral and Teaching Hospital, Mbale, Uganda; 7 Department of Obstetrics and Gynaecology, Busitema University, Tororo, Uganda; 8 Department of Medicine, Jinja Regional Referral Hospital, Jinja, Uganda; 9 Department of Medicine, Our Lady Health of the Sick, Nkozi Hospital, Nkozi, Uganda; 10 Covid Task Force Institution, Mengo Hospital, Kampala, Uganda; 11 Department of Medical Microbiology, Gulu University, Gulu, Uganda; 12 Department of Community and Public Health, Busitema Universiy, Tororo, Uganda; 13 Department of Medicine, Jaro Hospital, Wakiso, Uganda; 14 Department of Anatomy, Makerere University, Kampala, Uganda; 15 Department of Pediatrics and Child Health, Makerere University, Kampala, Uganda

**Keywords:** Prolonged hospitalization, Length of hospital stay, COVID-19

## Abstract

**Introduction:**

Identification of factors predicting prolonged hospitalization of patients with coronavirus disease (COVID-19) guides the planning, care and flow of patients in the COVID-19 Treatment Units (CTUs). We determined the length of hospital stay and factors associated with prolonged hospitalization among patients with COVID-19 at six CTUs in Uganda.

**Methods:**

We conducted a retrospective cohort study of patients admitted with COVID-19 between January and December 2021 in six CTUs in Uganda. We conducted generalized linear regression models of the binomial family with a log link and robust variance estimation to estimate risk ratios of selected exposure variables and prolonged hospitalization (defined as a hospital stay for 14 days or more). We also conducted negative binomial regression models with robust variance to estimate the rate ratios between selected exposures and hospitalization duration.

**Results:**

Data from 968 participants were analyzed. The median length of hospitalization was 5 (range: 1–89) days. A total of 136/968 (14.1%: 95% confidence interval (CI): 11.9–16.4%) patients had prolonged hospitalization. Hospitalization in a public facility (adjusted risk ratio (ARR) = 2.49, 95% CI: 1.65–3.76), critical COVID-19 severity scores (ARR = 3.24: 95% CI: 1.01–10.42), and malaria co-infection (adjusted incident rate ratio (AIRR) = 0.67: 95% CI: 0.55–0.83) were associated with prolonged hospitalization.

**Conclusion:**

One out of seven COVID-19 patients had prolonged hospitalization. Healthcare providers in public health facilities should watch out for unnecessary hospitalization. We encourage screening for possible co-morbidities such as malaria among patients admitted for COVID-19.

## Introduction

Prolonged hospitalization is often unnecessary [1, 2], and is associated with various complications (such as hospital-acquired infections) and increased costs [3]. Furthermore, prolonged hospital stay increases the indirect costs faced by the patients, compromises patient in-hospital experience, and makes hospital beds unavailable [4]. This is very important for a country such as Uganda, where we (the authors) witnessed many patients and loved ones die during the second wave of COVID-19 because they could not secure hospital beds. Predicting the length of hospital stay among COVID-19 patients guides proper planning for adequate bed capacity [5].

Studies conducted about the length of hospital stay among COVID-19 patients have shown that the median hospital length of stay among COVID-19 patients varies across countries [6–13]. Notably, a meta-analysis that included 52 studies revealed that the median length of hospital stay among COVID-19 patients was 14 days (Interquartile range (IQR) 10–19) in China and 5 days (IQR 3–9) in the United Kingdom [11]. Several factors are associated with prolonged hospitalization among COVID-19 patients including age, sex, serious COVID-19 illness, presence of co-morbidities, and patient-to-health worker ratio [6–9, 13–15].

However, there is limited information on the length of hospital stay among COVID-19 patients and its associated factors from sub-Saharan Africa. Information on the length of hospital stay could improve patient care, guide the planning for COVID-19 patients, and design of interventions to reduce hospital stay such as community care and follow-up of patients [16]. We assessed the hospitalization length and its associated factors among COVID-19 patients admitted at six health facilities in Uganda.

## Materials and methods

### Study design

This was a retrospective cohort study conducted between January and December 2021.

### Study setting

This study was conducted at six COVID-19 treatment units (CTUs) in Uganda. These include: two public hospitals (Moroto regional referral hospital and Jinja regional referral hospital), one private hospital; Jaro hospital, and three faith-based hospitals (Mengo hospital, Nsambya hospital, and Nkozi hospital).

### Participating hospitals

Moroto Hospital is a public hospital funded by the Uganda Ministry of Health (MOH) in Moroto district. Moroto Hospital has a 172-bed capacity; the COVID-19 treatment unit has a capacity of 50 beds. Treatment of COVID-19 is free of charge.

Jinja regional referral hospital is located in Jinja district, approximately 84 km East of the capital city, Kampala. It has a bed capacity of 600 and serves as a referral hospital for 10 districts. Treatment of COVID-19 is free of charge.

Jaro hospital is a private for-profit hospital located in Wakiso district with an approximately 30-bed capacity. Treatment of COVID-19 is at a fee.

Mengo hospital is a church-based private not-for-profit, oldest hospital in the country, having been established by missionaries in 1897 and is owned by the Church of Uganda. Mengo has a bed capacity of over 300 beds. The Mengo CTU is a 48-bed unit accredited by the MOH to provide treatment for moderate and severe cases of COVID-19. The hospital started as a holding center for COVID-19 in August 2020, actual treatment started in January 2021. Treatment of COVID-19 is at a fee.

Nkozi Hospital is a Private Not for Profit (PNFP) Hospital owned by Kampala Archdiocese. It was founded by the White Sisters of Our Lady of Africa from the Netherlands in 1942 in Mawokota County South, off the Kampala-Masaka Road. Nkozi hospital operates 24 h daily and offers curative, preventive, promotive, and referral services both at the static and in outreaches. It has both inpatient and outpatient services. It has a 100-bed capacity. Treatment of COVID-19 is at a fee.

Nsambya hospital is a private not-for-profit hospital located in the southern part of the capital city Kampala, about 3 km from the city center. Nsambya has an approximately 400-bed capacity hospital. Treatment of COVID-19 is at a fee.

### Study sample

We included data for all patients with a polymerase chain reaction (PCR) or rapid diagnostic test (RDT) or radiological confirmation of COVID-19 among patients hospitalized in the six hospitals above, from January 2021 to December 2021, and had data on length of hospitalization stay. We enrolled 968 participants and this provided absolute precision ranging from 0.9% to 3.1% for prolonged hospitalization estimates ranging from 2 to 50%, which we deemed adequate.

### Variables

The outcome variable was prolonged hospital stay. We defined prolonged hospital stay as admission lasting at least 14 days by following previous studies [17–20]. We also analyzed the length of hospitalization as a count variable denoting the number of days admitted. The independent variables included sociodemographic characteristics such as age, sex, marital status, and nationality, clinical factors such as COVID-19 vaccination, human immunodeficiency virus (HIV) status, history of comorbid conditions [hypertension, diabetes mellitus, tuberculosis and chronic obstructive pulmonary disease (COPD)], malaria test result, COVID-19 severity score and health system-related factors such as health facility category. Independent clinicians and trained research assistants extracted data from the patient files about demographics, clinical factors, and health system-related factors. COVID-19 severity score was defined as:

Mild: normal saturation, no need for intravenous treatment.

Moderate: normal saturation ≥ 94%, but in need of intravenous medication.

Severe/critical: saturation < 94%, and in need of oxygen therapy [21].

### Data collection

Data were extracted from patients’ files by trained research assistants, who were mostly COVID-19 treatment unit nurses using data collection entry forms designed and administered in KoBo Toolbox (Cambridge, MA, USA). KoBo Toolbox is open-source software developed by the Harvard Humanitarian Initiative with support from United Nations agencies, CISCO, and partners to support data management by researchers and humanitarian organizations (https://www.kobotoolbox.org/). All completed forms were uploaded onto KoBo Toolbox servers. These servers are secure and encrypted with strong safeguards and protection against data loss. Data were then exported into Microsoft Excel and Stata 17.0 for cleaning, coding, and analysis.

### Statistical analysis

Data were analyzed using Stata version 17.0 (StataCorp LLC, College Station, TX, USA). We summarized continuous variables using means with standard deviations or medians with interquartile ranges and categorical variables using frequencies and percentages. Fisher’s exact tests were used for the comparison of categorical variables. We conducted generalized linear regression models of the binomial family with a log link and robust variance estimation to estimate risk ratios of selected exposure variables and prolonged hospitalization, defined as being hospitalized for 14 days or more [17–20]. We also conducted negative binomial regression models with robust variance estimates to estimate the rate ratios between selected exposures and the number of days spent in the hospital. Our choice of a negative binomial regression model was made because there was evidence of over-dispersion (variance greater than the mean) with the Poisson regression model [22]. As such, we estimated the rate of hospitalization, defined as the number of days spent in the hospital per unit time (assumed to be 1 year in our study for ease of interpretation) [22].

## Results

### Participant characteristics

**Fig. 1:**
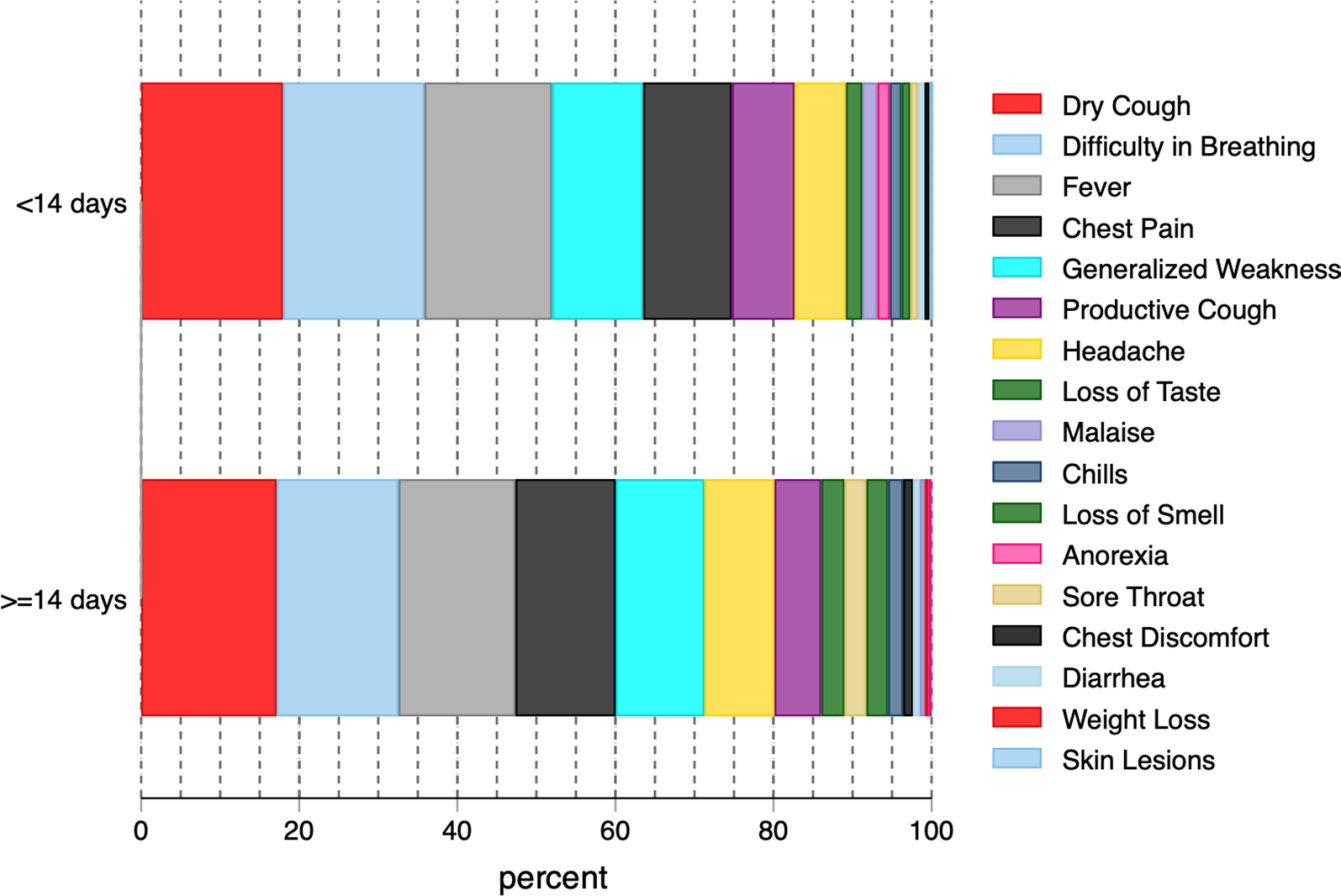
Symptoms comparison between patients hospitalized for 14 days or more and those who were hospitalized for less than 14 days.

A total of 968 COVID-19 patients were included in this study. The median age of participants was 52 years, interquartile range (IQR) was 34–67 years. Slightly more than half of the participants were female, 514/968 (53.2%). The median (IQR) time from symptom onset to admission was 6 days (4–10). The distribution of clinical signs and symptoms was similar between those with prolonged and those without prolonged hospitalization; however, participants with prolonged hospitalization had a higher proportion of headaches and chest pain. Further characteristics are shown in Table 1 and Fig. 1.

**Table 1:** Socio-demographic characteristics of patients with COVID-19 in Uganda

Variables	< 14 days (*n* = 832)	≥ 14 days (*n* = 136)	Total (968)
Age category (years)			
< 18	47 (5.65%)	9 (6.62%)	56 (5.79%)
18 to < 50	326 (39.18%)	56 (41.18%)	382 (39.46%)
50–	458 (55.05%)	71 (52.21%)	529 (54.65%)
Sex			
Female	437 (52.52%)	77 (56.62%)	514 (53.10%)
Male	394 (47.36%)	59 (43.38%)	453 (46.80%)
Health facility category			
PNFP	473 (56.85%)	54 (39.71%)	527 (54.44%)
Public hospital	356 (42.79%)	82 (60.29%)	438 (45.25%)
Private hospital	3 (0.36%)	0 (0.00%)	3 (0.31%)
Nationality			
Ugandan	817 (98.20%)	132 (97.06%)	949 (98.04%)
Others	15 (1.80%)	4 (2.94%)	19 (1.96%)
Marital status			
Married/cohabiting	570 (68.51%)	94 (69.12%)	664 (68.60%)
Single/divorced	159 (19.11%)	19 (13.97%)	178 (18.39%)
COVID-19 vaccination			
No	823 (98.92%)	135 (99.26%)	958 (98.97%)
Yes	9 (1.08%)	1 (0.74%)	10 (1.03%)
Highest level of education			
None	65 (7.81%)	17 (12.50%)	82 (8.47%)
Primary	42 (5.05%)	19 (13.97%)	61 (6.30%)
Secondary	71 (8.53%)	15 (11.03%)	86 (8.88%)
Tertiary	168 (20.19%)	33 (24.26%)	201 (20.76%)
HIV			
No	804 (96.63%)	129 (94.85%)	933 (96.38%)
Yes	28 (3.37%)	7 (5.15%)	35 (3.62%)
Hypertension history			
No	587 (70.55%)	101 (74.26%)	688 (71.07%)
Yes	245 (29.45%)	35 (25.74%)	280 (28.93%)
Diabetes history			
No	695 (83.53%)	120 (88.24%)	815 (84.19%)
Yes	137 (16.47%)	16 (11.76%)	153 (15.81%)
COPD history			
No	831 (99.88%)	136 (100.00%)	967 (99.90%)
Yes	1 (0.12%)	0 (0.00%)	1 (0.10%)
TB history			
No	812 (97.60%)	130 (95.59%)	942 (97.31%)
Yes	20 (2.40%)	6 (4.41%)	26 (2.69%)
Malaria test result			
Negative	769 (92.43%)	129 (94.85%)	898 (92.77%)
Positive	63 (7.57%)	7 (5.15%)	70 (7.23%)
COVID-19 severity score			
Mild	64 (7.69%)	4 (2.94%)	68 (7.02%)
Moderate	350 (42.07%)	64 (47.06%)	414 (42.77%)
Severe	382 (45.91%)	63 (46.32%)	445 (45.97%)
Critical	35 (4.21%)	5 (3.68%)	40 (4.13%)

### Prolonged hospitalization

**Fig. 2:**
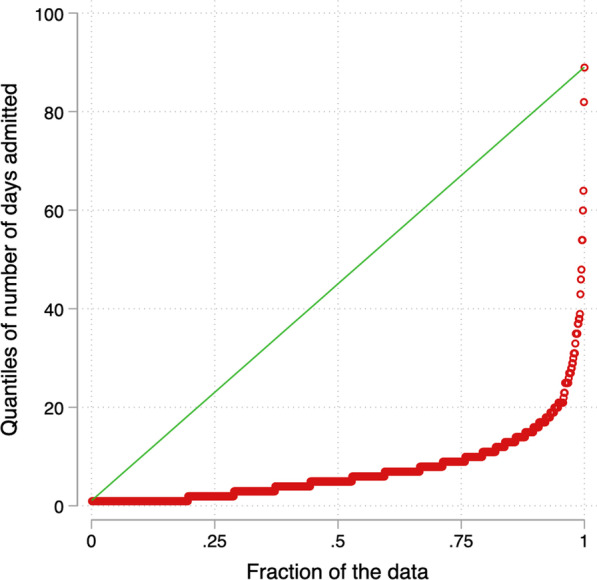
Quantile plot showing days of hospitalization of COVID-19 patients in six hospitals in Uganda

The median length of hospitalization was 5 days (IQR: 2–9 days). The minimum number of days admitted was one day and the maximum number of days admitted was 89 days. A total of 136/968 (14.1%: 95% CI: 11.9–16.4%) patients were admitted for 14 days or longer. Of the patients admitted 14 days or longer, 20/136 (14.7%) died, compared to 199/832 (23.9%) admitted for less than 14 days as shown in Fig. 2.

### Factors associated with prolonged hospitalization

Patients in government facilities were 2.5 times more likely to spend 14 days or more under admission compared to patients admitted in private health care facilities [adjusted risk ratio (ARR = 2.49: 95% CI: 1.65–3.76, P < 0.001)]. Patients admitted with severe/critical COVID-19 severity scores were 3 times as likely to spend 14 days or more under admission compared to patients with mild COVID-19 severity scores (ARR = 3.24: 95% CI: 1.01–10.42, P = 0.049).

The rates of hospitalization were higher among patients admitted to public (government) health facilities compared to patients admitted to private health care facilities [adjusted incident rate ratio (AIRR = 1.82 95% CI: 1.56–2.12, P < 0.001)]. Patients admitted with severe/critical disease had higher rates of hospitalization compared to patients admitted with mild COVID-19 disease (AIRR = 1.83: 95% CI, 1.46–2.28, P < 0.001). Patients with a positive malaria test had lower rates of hospitalization (hospital stay) compared to patients with a negative malaria test (AIRR = 0.67: 95% CI: 0.55–0.83) as shown in Table 2.

**Table 2:** Factors associated with prolonged hospitalization among patients with COVID-19 in Uganda

Variable	CRR	P-value	ARR* [95% CI]	P-value	AIRR** [95% CI]	P-value
Age category (years)						
< 18	1		1		1	
18 to < 50	0.91 [0.48, 1.74]	0.78	0.57 [0.25, 1.33]	0.195	0.79 [0.60, 1.05]	0.104
≥ 50	0.84 [0.44, 1.58]	0.579	0.55 [0.22, 1.34]	0.189	0.76 [0.56, 1.03]	0.077
Sex						
Female	1		1		1	
Male	0.87 [0.63, 1.19]	0.384	0.82 [0.58, 1.16]	0.263	0.92 [0.81, 1.06]	0.249
Health facility category						
Private/PNFP	1		1		1	
Public	1.84 [1.33, 2.53]	< 0.001	2.49 [1.65, 3.76]	< 0.001	1.82 [1.56, 2.12]	< 0.001
Nationality						
Ugandan	1		1		1	
Others	1.51 [0.62, 3.67]	0.359	2.27 [0.99, 5.22]	0.053	1.47 [0.67, 3.19]	0.334
Marital status						
Married/cohabiting	1		1		1	
Single/divorced	0.75 [0.47, 1.20]	0.234	0.57 [0.31, 1.05]	0.073	0.82 [0.69, 0.98]	0.025
COVID-19 vaccination						
No	1		1			
Yes	0.71 [0.11, 4.59]	0.719	1.11 [0.16, 7.55]	0.916	1.00 [0.68, 1.46]	0.995
HIV						
No	1		1			
Yes	1.45 [0.73, 2.86]	0.289	1.68 [0.66, 4.28]	0.281	0.90 [0.57, 1.43]	0.661
Hypertension history						
No	1		1			
Yes	0.85 [0.59, 1.22]	0.38	0.91 [0.59, 1.41]	0.666	1.04 [0.87, 1.23]	0.699
Diabetes mellitus history						
No	1		1			
Yes	0.71 [0.43, 1.16]	0.173	0.56 [0.27, 1.15]	0.114	0.85 [0.66, 1.10]	0.222
Tuberculosis history						
No	1		1			
Yes	1.67 [0.81, 3.44]	0.162	1.13 [0.52, 2.48]	0.751	0.96 [0.71, 1.32]	0.821
Malaria test						
Negative	1		1			
Positive	0.70 [0.34, 1.43]	0.325	0.55 [0.26, 1.16]	0.118	0.67 [0.55, 0.83]	< 0.001
COVID-19 severity score						
Mild	1		1			
Moderate	2.63 [0.99, 6.99]	0.053	2.72 [0.87, 8.48]	0.084	1.63 [1.31, 2.03]	< 0.001
Severe/critical	2.38 [0.90, 6.33]	0.081	3.24 [1.01, 10.42]	0.049	1.83 [1.46, 2.28]	< 0.001

*ARR *adjusted risk ratio, *AIRR* adjusted incident rate ratio

## Discussion

Our study showed that the median length of hospital stay among patients with COVID-19 hospitalized at six CTUs in Uganda was 5 days, with an interquartile range of 2 to 9 days. A meta-analysis including 52 studies revealed similar findings in developed countries [11]. Our findings are also similar to findings from Saudi Arabia [(6 days) [9], (7 days) [8]], London (6 days) [23], the United States (6 days) [10], France (9 days) [12], and Tunisia (8 days) [13]. However, the median length of hospital stay in our study is higher than that reported in Australia (3 days) [19]. The difference could have resulted from the efficiency of the health system in Australia which could have improved the quality of care. Furthermore, health-seeking behavior and availability of testing services were probably much better in Australia which ensured that patients presented with less severe disease and hence did not need prolonged hospitalization.

Nevertheless, the median length of hospital stay in our study is lower than that reported in Ethiopia (12 days) [6] and studies conducted in China [(13 days) [20] (17 days) [7] (14 days) [11]]. The differences could have been due to demographic differences. For instance, in Wu’s study in China, co-morbidities were more common, with 30% (38/125) reported to have diabetes compared to 16% (153/968) in our study, and 46% (57/125) reported to have hypertension compared to 29% (280/968) in our study [20]. Additionally, there could be differences in the guidelines for admission and discharge between countries and the timing of the studies within the pandemic.

Patients with severe/critical disease spent more time in the hospital. This was not surprising and was probably related to lung damage associated with more severe forms of the disease [24]. Our findings are similar to those observed by Kacem et al*.* in Tunisia [13]. Patients with severe/critical disease required respiratory support or oxygen for longer periods, which was not available in home management.

Patients spent longer in public hospitals compared to private hospitals. This could be related to the high patient-to-health-worker ratio and limited specialized care in public hospitals. Also, since care in public health facilities was free, patients likely waited until full recovery to accept discharge from the health facility. In many private health facilities, it was common for patients to request transfer to public health facilities or to request discharge to reduce costs. Similarly, a study conducted in Brazil also found that public health care was associated with prolonged hospitalization among COVID-19 patients [15].

Patients with a positive malaria test spent less time in the hospital compared to patients with a negative malaria test. Since the signs and symptoms of malaria overlap with COVID-19, it is likely that the primary cause of ailment in some COVID-19–malaria co-infected patients was malaria, and upon receipt of treatment, rapid improvement occurred as is characteristic of malaria episodes. The alternative explanation could be that these patients died faster, but we do not see evidence of that effect in our data.

### Strength and limitations

This was a multicenter study including public, private, and private not-for-profit hospitals in both urban and rural settings thus our results are representative of the bigger population of COVID-19 patients treated in the country. We also believe that our sample size was powered enough to detect potential associations between variables of interest. However, due to the retrospective nature of the study, we were not able to obtain all data concerning the variables of interest. There was also a challenge of some files having missing information.

## Conclusion

One out of seven COVID-19 patients had prolonged hospitalization. Healthcare providers in public health facilities should watch out for unnecessary hospitalization. We encourage screening for possible co-morbidities such as malaria among patients admitted for COVID-19.

## Data Availability

The datasets used and/or analyzed during the current study are available from the corresponding author on reasonable request.

## Abbreviations

COVID-19: Coronavirus disease 2019
AIDS: Acquired immunodeficiency syndrome
HIV: Human immunodeficiency virus
WHO: World Health Organization
PCR: Polymerase chain reaction
RDT: Rapid diagnostic test
IQR: Interquartile range
AFT: Accelerated failure time
